# Past Negative Consequences of Unnecessary Delay as a Marker of Procrastination

**DOI:** 10.3389/fpsyg.2022.787337

**Published:** 2022-02-21

**Authors:** Frode Svartdal, Efim Nemtcan

**Affiliations:** Department of Psychology, UiT the Arctic University of Norway, Tromsø, Norway

**Keywords:** procrastination, maladaptive delay, irrational delay, procrastination scale, strategic delay

## Abstract

Standard definitions of procrastination underscore the irrational nature of this habit, a critical criterion being that the procrastinating individual delays despite expecting to be worse off for the delay. However, an examination of more than 175 items in 18 procrastination scales reveals that they do not address such a forward-looking criterion. Consequently, scales run the risk of not separating maladaptive and irrational delays from other forms of delay. We propose that forward-looking considerations may not be the best way of operationalizing the irrationality involved in procrastination and argue that scales should instead focus on past negative consequences of unnecessary delay. We suggest a new scale to measure such procrastination-related negative consequences and demonstrate that this scale, used separately or combined with established procrastination scales, performs better in predicting negative states and correlates to procrastination than established scales. The new scale seems to be helpful in separating trivial forms of unnecessary delay from maladaptive forms and hence represents a potentially valuable tool in research and clinical/applied efforts.

## Introduction

Behavioral delay is a core characteristic of procrastination. However, as some forms of delay are rational and sensible (e.g., wait until tomorrow to mow your lawn because it is raining today), the delays seen in procrastination are defined as those chosen despite the individual realizing or expecting to be worse off for the delay (Lay, 1986; Steel, 2007; Ferrari, 2010; Klingsieck, 2013). For example, Klingsieck (2013) compared strategic delay and procrastination. Both are characterized by an action being *delayed*, by an action being *intended*, by involving acts that are *necessary or of personal importance*, and by acts being *voluntarily chosen*. In contrast to strategic delay, procrastination is *unnecessary* or *irrational*, is *chosen despite being aware of its potentially negative consequences*, and is *accompanied by discomfort or other negative consequences*.^1^ Hence, given subjective norms and cognitive-affective evaluations (Milgram and Naaman, 1996; van Eerde, 2003), procrastinatory behavior is seen as “irrational” or dysfunctional in the sense that the individual chooses to put off against better judgment (e.g., Andreou and White, 2010).

Given this understanding of procrastination, one might expect procrastination scales to include items measuring forward-looking considerations of potential negative consequences of putting off. However, they do not. Examination of more than 175 items in 18 scales reveals that only two items address some form of forward-looking cost calculation of negative consequences of procrastination. The first is item 13 from the Tuckman (1991) procrastination scale, “Even though I hate myself if I don’t get started, it doesn’t get me going.” The second is item 17 from the Academic Procrastination State Inventory (APSI; Schouwenburg, 1995), which refers to fear of failure as a reason for putting off. As a result, existing scales run the risk of being indiscriminate in assessing procrastination. For example, if trivial delays are incorrectly perceived as procrastination because of harsh self-judgment (Sirois, 2014; Svartdal and Steel, 2017), procrastination scale scores may be inflated. As existing procrastination scales are the tools used to assess the relation of procrastination to negative states and outcomes (e.g., stress, anxiety, lack of energy, depression, reduced self-efficacy, and well-being; for reviews and meta-analyses, see van Eerde, 2003; Steel, 2007; Klingsieck, 2013) as well as prevalence estimates (e.g., Ferrari et al., 2005), care should be taken to assess the quality of such scales. Hence, scale items assessing the maladaptive and irrational aspects of procrastination are of great scientific and practical interest.

### Forward-Looking Considerations of Negative Consequences

Given the importance of the “expecting to be worse off” and “act against better judgment” criteria for identifying the maladaptive and irrational side of procrastinatory behavior, a simple solution might be to include items that reflect consideration of future disadvantages associated with putting off. However, we argue that this solution may not be viable because (1) the subjective availability of future negative consequences of putting off is questionable, (2) studies of subjective reasons for procrastination do not support the existence of forward-looking cost considerations, and (3) known mechanisms in procrastination do not appear to involve future cost considerations.

#### Subjective Availability and Consideration of Future Negative Consequences

First, one may ask what kind of negative consequences people might have in mind when deciding to put off. Although reviews and meta-analyses have amply documented detrimental correlates to, or consequences of, procrastination (van Eerde, 2003; Steel, 2007), it is unclear whether such correlates or consequences are subjectively available to procrastinators when choosing to delay unnecessarily. For example, procrastination is related to increased stress (Tice and Baumeister, 1997; Sirois, 2014), which is understandable as delayed work with deadlines must be completed in a shorter time. Hence, increased stress might be a subjectively anticipated consequence speaking against putting off tasks. However, we are not aware of research documenting procrastinators to opt to delay despite expecting increased stress. To our knowledge, a similar conclusion applies to many, maybe most, of the observed correlations between procrastination and adverse states and outcomes. For example, although research has documented a reliable and moderately negative relation between academic performance and procrastination (see Kim and Seo, 2015, for meta-analysis), it is not apparent that an individual chooses to procrastinate despite expecting to obtain lower grades. Similarly, research has demonstrated a correlation between procrastination and depression and anxiety (Ferrari et al., 1995; Stöber and Joormann, 2001), but it is not known (or even likely) that the individual has increased depression or anxiety in mind when deciding to procrastinate.

Studies that have examined subjective reasons for putting off planned work (e.g., Schraw et al., 2007) provide little evidence to support forward-looking considerations of negative consequences as part of the decision to put off. For example, one candidate might be *fear of failure* – putting off work on a task because of a lack of perceived competence to complete the task successfully (e.g., Milgram et al., 1988). Although fear of failure may lead to delayed task execution, the opposite has also been reported, as fear of failure may inspire increased motivation and makes one start earlier and work harder (Schraw et al., 2007, p. 19). Conceptually, fear of failure does not create delays “despite expecting to be worse off” either. On the contrary, fear of failure may create delays to protect the individual from doing things with a high probability of failing, which may be a rational decision seen from the actor’s perspective when choosing to delay. It is not surprising, therefore, that the correlation between procrastination and fear of failure is low, *r* = 0.18 (Steel, 2007). Other studies (e.g., Lindblom-Ylänne et al., 2015) have identified forward-looking considerations in strategic delay, which (by definition) is not procrastination. Grunschel et al. (2013) had students rate their reasons for academic delay using 14 items, two of which addressed future considerations, but in a *positive* and strategic sense (“Belief that one works better under high pressure” and “Anticipation of a better opportunity”).

#### Mechanisms in Procrastination Are Not Likely to Involve Future Cost Considerations

Given the nature of procrastinatory behavior as impulsive and maladaptive deviations from plans with limited future temporal orientation (Specter and Ferrari, 2000; Díaz-Morales and Ferrari, 2015; Sirois and Pychyl, 2018), it is likely that procrastinatory behavior often may result without much consideration of potential negative consequences. First, procrastinators may, through rationalization and wishful thinking, perceive their procrastinatory behavior as rational. Thus, “irrational” decisions to put off may subjectively appear as rational when decisions are made (e.g., Sigall et al., 2000; Tuckman, 2005). In these cases, individuals put off with no or little concern for future negative consequences. Second, future episodic thinking is negatively related to procrastination (Rebetez et al., 2016), indicating that procrastinators are less likely than others to consider future negative consequences when deciding to put off. Third, as potential negative consequences of putting off may be temporally distant, they tend to have little weight in cost-benefit considerations for action here and now (e.g., Temporal Motivation Theory, TMT; Steel and König, 2006; Gröpel and Steel, 2008). This is the case particularly for procrastinators, who are impulsive and present-oriented (e.g., Sirois and Pychyl, 2013). Fourth, the fact that decisions to put off are made intuitively, embedded in the flow of action and in the presence of temptations and distractions (e.g., Steel et al., 2018) pinpoints procrastination as a breakdown in self-regulation (Steel, 2007) rather than an outcome of a cost-benefit analysis. Fifth, as putting off is likely when working with aversive and boring tasks, emotional regulation – “giving in to feel good” – is a well-documented mechanism. This mechanism gains importance as individuals get tired, are low in energy, or are stressed (Tice and Baumeister, 1997; Sirois and Giguère, 2018), all suboptimal states to make decisions.

In conclusion, forward-looking considerations of future negative consequences of procrastinatory behaviors seem to be highly problematic in identifying the maladaptive nature of procrastination. The criterion is definitional but not sufficiently supported by theory or empirical studies.

### Past Negative Consequences of Procrastination as a Criterion of Maladaptive Delay

If forward-looking considerations of negative consequences of delayed actions are problematic in identifying the maladaptive nature of procrastinatory behavior, then what? In this paper, we propose an alternative view, one that emphasizes subjectively experienced past negative consequences of unnecessary delay as an important marker of the maladaptive and irrational side of procrastination. We suggest a new scale to measure such procrastination-related past negative consequences and argue that this scale, used separately or combined with established procrastination scales, may be superior to existing scales in identifying the problematic and maladaptive aspects of procrastination. Except for the Procrastination Assessment Scale for Students (PASS; Solomon and Rothblum, 1984), self-report procrastination scales do not include items to assess discomfort or negative feelings associated with procrastinatory episodes. Clearly, a scale that differentiates maladaptive and trivial forms of unnecessary delay is of great interest, both in research and in applied/clinical settings.

#### Theoretical and Empirical Basis

As noted, the forward-looking criterion for procrastination (“…delay despite expecting to be worse off for the delay”) is definitional, with minimal explicit theoretical or empirical foundation. Turning this criterion to past negative consequences of unnecessary delay, the definition is kept unchanged, except that the “worse off”-criterion points to past experience rather than future expectations. Empirically, a retrospective criterion is indeed meaningful, as discomfort or and subjective negative feelings associated with procrastinatory behavior have been pointed out by multiple authors in the field (e.g., Ellis and Knaus, 1977; Solomon and Rothblum, 1984; Ferrari, 1998; Simpson and Pychyl, 2009; Klingsieck, 2013). Furthermore, a retrospective criterion with a focus on the discomfort and negative feelings associated with unnecessary delay is consistent with a self-regulation perspective on procrastination (e.g., Tice and Bratslavsky, 2000). Procrastination is assumed to be a breakdown in self-regulation (Steel, 2007), and attempts to exercise self-control is associated with negative emotions, as is failure to self-regulate itself (Tice and Bratslavsky, 2000; Tice et al., 2001; Sirois and Pychyl, 2013). Thus, unnecessary delays related to self-regulation failure should be characterized by discomfort and subjective negative feelings (Krause and Freund, 2014). When such self-regulation failures become vivid to the person in the sense that they are pointed out by others, accompanied by loss, or in other ways demonstrate that one is worse off because of the delay, we find it likely that they are perceived and remembered in a better way compared to delays that have no specific consequences. Hence, negative consequences associated with procrastination have an important discriminative function that helps distinguish it from other forms of delay (e.g., strategic delay, rational forms of delay) and even from procrastination with no specific negative consequences. For example, Lindblom-Ylänne et al. (2015) noted that a subgroup of procrastinators in their study did not demonstrate any subjective discomfort associated with their unnecessary delay, indicating that subjective discomfort and negative feelings associated with procrastination may be an indicator of maladaptive forms.

Importantly, some negative consequences are embedded in short-term positive consequences. Procrastination is regarded as a self-regulation failure with short-term mood repair and emotion regulation as important ingredients (e.g., Tice and Bratslavsky, 2000; Sirois and Pychyl, 2013; Bytamar et al., 2020). Short-term mood repair and emotion regulation imply that negative emotions and cognitions are important antecedents for procrastinatory episodes and that procrastination works to alleviate these negative emotions/thoughts. This view may appear as exactly the opposite of the view discussed in the present paper (i.e., that procrastination is followed by negative consequences). However, it must be remembered that mood repair and emotional regulation both indicate a self-regulation failure. Thus, the immediate positive effect brought about by mood repair is positive only for a limited time, and it is likely that the individual, even at the moment of putting off, or later, experiences discomfort or other negative consequences (see Sirois and Pychyl, 2013, p. 117). A scale focusing on the negative consequences of procrastination must keep this positive-negative duality of procrastinatory episodes in mind.

##### Immediate Negative Consequences

Discomfort and negative consequences of procrastination may be immediate and delayed, and both forms should be available for self-report. *Immediate negative consequences* of procrastination (e.g., social sanctions from others; realization that the delay was unwise) must be assumed to be more vivid compared to forms of procrastination that do not evoke any specific consequences (e.g., skip working on a difficult assignment with no immediate consequence). Importantly, research has documented that subjective vividness enhances memory (e.g., Kensinger, 2007). Hence, self-report of procrastinatory behaviors associated with negative emotions (i.e., the scale suggested in this paper) should be more accurate compared to self-report of procrastination in general (i.e., a standard procrastination scale).

##### Delayed Negative Consequences

A retrospective focus opens for a broader understanding of “negative consequences.” Some negative consequences may become apparent only in a retrospective evaluation. For example, after putting off important work, you may realize the next day (or even later) that the delay was unfortunate and hence feel regret and formulate negative cognitions (e.g., Stainton et al., 2000). In their classic study, Tice and Baumeister (1997) demonstrated that the early parts of the semester appear as stress-free and pleasant for procrastinating students, as putting off academic work has no or minimal immediate negative consequences. However, the benefits of procrastination early in the semester had negative consequences later when the student had to work harder to catch up, with more stress and illness as predictable consequences. This indicates that a scale intended to measure negative consequences of procrastination must address a sufficiently long-time span.

For both immediate and delayed negative consequences, an obvious advantage of retrospective analysis is that such an analysis may probe the negative consequences of procrastination over various domains and situations. A scale probing negative consequences of procrastination should assess consequences across several domains/situations in terms of frequency (in which domains/situations are negative consequences most often reported?) as well as relative importance (in which domains/situations do negative consequences affect the individual the most)? If negative consequences of procrastination address the problematic core features of procrastination, such information is of prime importance.

### Prior Research on Past Negative Consequences of Procrastination

Whereas prior research has examined reasons for why people procrastinate (e.g., Solomon and Rothblum, 1984), little research has focused on the specific subjective negative consequences associated with procrastination (see Day et al., 2000, p. 127, for an exception). This is surprising, as procrastination research has provided ample evidence of an association between procrastination and adverse states and consequences associated with this habit (van Eerde, 2003; Steel, 2007; Sirois and Kitner, 2015). However, the fact that procrastination subjectively may be positive in the short-term perspective but harmful in the long run, pinpointing the consequences of unnecessary delay may sometimes be complex and possible only in a longer retrospective time frame. Another factor explaining the relative lack of research on subjectively perceived negative consequences of procrastination is the interplay between subjective values and criteria for unnecessary delay. For example, Grund and Fries (2018) demonstrated that high procrastinators tend to be low in achievement values and high in values related to well-being (Study 1), and that people favoring conservative values are more likely to perceive academic procrastination as a failure, whereas individuals endorsing liberal values were more likely to consider situational factors of procrastination (Study 3). Therefore, subjective values may blur the distinction between maladaptive and acceptable forms of delay.

We identified three areas of research that relate directly to negative consequences of procrastination and subsequent procrastination-relevant thinking and behavior. First, in counterfactual thinking (e.g., Roese, 1997), the individual cognitively simulates alternatives to factual states of affairs. Such simulations may compare factual outcomes to better alternatives (upward counterfactuals, e.g., “if I had worked harder, I would have passed with a better grade” when receiving a disappointing grade) or to worse alternatives (downward counterfactuals, e.g., “at least I did not fail the exam” when receiving the disappointing grade). Whereas downward counterfactuals may act as a strategy to repair disappointment and protect the self with little motivation to change, upward counterfactuals generate thoughts about alternative ways of action and may therefore inspire change in the future. Not surprisingly, Sirois (2004) found support for procrastination to be linked to downward counterfactuals, with an immediate positive effect of protecting self-image at the cost of not exploring possible change in the future. Second, research on self-forgiveness in procrastination relates directly to the negative consequences of procrastination and demonstrates how the cognitive processing of such experiences may positively or negatively affect the individual. For example, Wohl et al. (2010) showed that self-forgiveness, the reduction in negative affect associated with procrastination, is positive in the sense that it reduces future procrastination. Similarly, Sirois (2014) found that procrastination is associated with lower levels of self-compassion and that lower levels of self-compassion at least in part explained the procrastination–stress relationship. Third, research addressing cognitions related to procrastination may also be relevant for the present research. Stainton et al. (2000) developed a scale, the Procrastination Cognitions Inventory (PCI), that contains a variety of statements related to own procrastination (e.g., item 1 “Why can’t I do what I should be doing?” and item 2 “I need to start earlier”). Importantly, Stainton et al. (2000, Study 2) administered this scale to measure past thoughts (last 3 weeks) as well as future thoughts (future 3 weeks) related to procrastination and found both to correlate moderately with trait procrastination, *r* = 0.54 and 0.48. In effect, these results^2^, as well as a subsequent study by Flett et al. (2012), indicate that negative cognitions about past procrastination are related to increased rumination, worry, distress, and stress.

Unfortunately, none of these contributions are reflected in general or academic procrastination scales. However, they support the present work in the sense that some negative consequences of past procrastination may affect future procrastination differently, depending on how they are handled (e.g., downward counterfactual thoughts; self-forgiveness). Others (e.g., negative thoughts related to procrastination) may act in a more direct way to foster future procrastination by increasing negative cognitions and emotions. Clearly, such information is valuable in scale development and interpretation of scale scores.

### Scale to Measure Negative Consequences of Procrastination

At present, there is no scale addressing the perceived negative consequences of procrastination. As procrastination-related negative consequences may occur in different domains and situations, the first step in developing such a scale is determining relevant domains/situations. Prior research (e.g., Gröpel and Kuhl, 2006; Ferrari et al., 2009; Klingsieck, 2013; Goroshit et al., 2020) has identified procrastination in several life domains, such as work (including academic work), everyday routines and obligations, health, leisure, family, and partnership, social and financial. Reviews and meta-analyses (e.g., van Eerde, 2003; Steel, 2007) have demonstrated procrastination tendencies (“trait procrastination”) to be relatively stable across domains and situations, but it is important to recognize that situational and personality variables may be important in facilitating or hindering actual instances of procrastinatory behavior from occurring (e.g., Wäschle et al., 2014; Steel and Klingsieck, 2016; Svartdal et al., 2020). Hence, we included items to cover procrastination in three different situations/domains that are important and relevant for students and the general population. In addition, we probed more general negative feelings and cognitions associated with procrastinatory episodes^3^.

#### Social

Social aspects of procrastination have received relatively little attention in the procrastination literature (Klingsieck, 2013). However, in several papers, Ferrari and colleagues have documented the role of social factors in procrastination. For example, social comparison is important among students (Ferrari and Patel, 2004), and procrastinators seem to be particularly sensitive to negative social information, probably to protect their self-image (Ferrari, 1991). Research also indicates that social norms and negative emotions associated with transgressing those norms are involved in procrastination (e.g., Giguère et al., 2016). Negative social consequences of procrastination may therefore be markers of problematic delay. Such consequences may appear in many forms, for example, when the procrastinating individual does not meet obligations in interaction with others and is confronted with that fact (e.g., “My friends complain that I delay things unnecessarily”).

#### Performance/Stress

As discussed, research evidence shows a reliable and moderately negative relation between academic performance and procrastination (Kim and Seo, 2015). The mechanisms involved are not clear. However, two classic performance-related negative consequences appear when unnecessary delay renders less time available for task completion and when the delay implies that one gets behind in work (e.g., Steel et al., 2018). Importantly, in both cases, reduced performance may not be apparent to the individual at the time of delay (or even at repeated occurrences of delay) but may become apparent in a retrospective evaluation. Possible scale items to measure this type of negative consequences could be “As a consequence of my tendency to delay things, I am behind in schoolwork” or “Because I delayed work, I must work under time pressure.”

#### Financial

Another set of consequences relates to financial loss or cost due to procrastination. Procrastinators are impulsive, and impulsive decisions and behaviors are associated with problems with personal finances (for an overview, see Gamst-Klaussen et al., 2019). Ferrari et al. (2009) demonstrated that life regret within the financial domain correlated moderately (*r* = 0.21–0.23) with procrastination score, higher compared to all other domains examined. Financial loss or cost due to procrastination may often be relatively easy to detect and report (e.g., item 15 in the AIP scale, “putting things off till the last minute has cost me money in the past” (Adult Inventory of Procrastination Scale; McCown et al., 1989). Other forms may be more subtle. For example, Reuben et al. (2015) demonstrated that procrastinating students were not only impulsive and preferred a smaller reward now compared to a larger reward 2 weeks later but also slow in cashing in their reward checks. In this case, financial loss may not be directly detectable by the procrastinator, but in retrospect, the maladaptive and irrational nature of such choices may become more visible. Hence, the financial loss/cost dimension was included in the scale.

#### Negative Emotions

It is well documented that negative emotions are potentially powerful drivers of procrastinatory behavior, as delay may be instrumental in mood repair and avoidance of aversive events (e.g., Blunt and Pychyl, 2000; Wohl et al., 2010; Pollack and Herres, 2020). The emotions of *shame, guilt*, or *regret* address negative feelings related to past events and are of particular interest in the present context. Shame and guilt are associated with transgression of social norms, and both emotions seem to be important in procrastination (Giguère et al., 2016). Lee and Hall (2020) reported a correlation of *r* = 0.36 between procrastination tendencies and shame, guilt, or regret in undergraduates. These authors also indicated that the negative emotions of guilt, shame, and regret loaded similarly to the latent factor “negative emotions.” In the present study, we included an item to address such “retrospective” negative emotions specifically.

#### Negative Cognitions

As discussed, the Procrastinatory Cognitions Inventory (PCI; Stainton et al., 2000) demonstrates that negative cognitions are positively correlated with procrastination. Several of the items in this scale address disappointment (e.g., item 5, “No matter how much I try, I still put things off”; item 10, “I am letting myself down”) and comparative dissatisfaction (item 6, “People want me to work and study more”). Student life offers many arenas where private standards may be challenged, such as exams, comparison to fellow students, and others. In the present studies, two items addressed this issue, one performance-related (working more slowly than others) and one related to negative thoughts in failed goal attainment due to procrastination (disappointment).

Table 1 summarizes the three situations and domains in which negative consequences of procrastination may appear, as well as negative cognitions and feelings related to procrastinatory episodes. Note that the situations/domains indicated in the table are suggestive and not exhaustive. If the negative consequences of procrastinatory behaviors define the troublesome aspect of this habit, a scale focusing on such consequences is likely to be useful in predicting the negative states and outcomes associated with procrastination. Thus, the scale suggested in this paper, the Negative Consequences of Procrastination (NCP), should be expected to predict known relations between procrastination and positive or negative states and outcomes with better precision compared to standard procrastination scales.

**TABLE 1:** Negative consequences of procrastination in different domains/situations.

	**Negative consequences of unnecessary delay**
Social	Negative social reactions from others
Loss, cost	Lost opportunities; financial loss; financial cost
Performance, stress	Less time for task completion; stress; get behind in academic work
Negative emotions	Shame, regret, guilt, worry associated with procrastination
Negative cognitions	Expected goals/standards not attained (disappointment)

## Study 1

The main purpose of Study 1 was to explore the utility of a scale, the NCP scale, using items that probe NCP over the situations/domains shown in Table 1, in a student sample. The expectation for this scale was that it, despite covering several different situations/domains, still conformed to a unidimensional construct. Moreover, as the NCP is more restrictive compared to standard procrastination scales, the overall mean score of this scale should be lower. The NCP scores should also correlate moderately to highly with a standard procrastination scale, as NCP depend on instances of procrastinatory behavior.

The respondents also answered a standard procrastination scale, the Irrational Procrastination Scale (IPS; Steel, 2010), as well as scales addressing well-being, lack of energy (LoE), and social loafing. Well-being was measured by the Satisfaction With Life Scale (SWLS) (Diener et al., 1985). Several studies have reported a negative relationship between well-being and procrastination (e.g., Sirois et al., 2003; Sirois, 2007; Beutel et al., 2016; Svartdal et al., 2016). In the present study, we expected a similar finding, but we expected that the NCP scale would be superior to the IPS in predicting subjective well-being.

As for LoE, Gröpel and Steel (2008) reported a strong correlation, *r* = 0.60, between procrastination and energy level in a large sample of 9,351 participants, a finding later repeated by Steel et al. (2018) in a student sample. Although the directional relationship between these constructs is not determined, LoE may both act as an antecedent factor in procrastination and as a consequence. For example, low energy increases the likelihood that work becomes aversive, and as task aversiveness is a strong predictor of procrastination (e.g., Blunt and Pychyl, 2000; Grunschel et al., 2013; Laybourn et al., 2019), procrastination may result. However, working with difficult tasks (e.g., academic tasks) may itself be more energy-demanding compared to working with simpler tasks, speaking for a reversal of the causal chain. In both cases, LoE should be associated with negative affect (e.g., Maslach and Jackson, 1984). Therefore, as the NCP scale addresses negative associated with procrastinatory episodes, we expected that the NCP scale would outperform the IPS in predicting LoE.

Well-being and LoE address general phenomena that must be expected to manifest themselves over various situations/domains. In contrast, social loafing is a phenomenon related to the social domain specifically. Despite being a thoroughly studied phenomenon (see Karau and Williams, 1993), the relationship between social loafing and procrastination is not much explored. Ferrari and Pychyl (2012) pointed out that social loafing and procrastination share similarities. For example, both constructs imply reduced motivation to engage in goal-directed task activities and reduced commitment to oneself (procrastination) or to others (social loafing). A difference is that procrastination is seen as an individual problem, whereas social loafing is observed in groups where loafer transgresses social norms and negatively affects group work (e.g., George, 1996). Given the similarities between these phenomena, a moderate correlation between perceived social loafing and procrastination, *r* = 0.30–0.45, was reported (Ferrari and Pychyl, 2012). In the present study, we assessed self-rated social loafing, expecting a similar relation to procrastination. As both procrastination and social loafing are maladaptive, and transgressing social norms is associated with negative emotions (Giguère et al., 2016), we expected that a scale that focuses explicitly on the maladaptive and negative aspects of procrastination – the NCP – will be superior in predicting social loafing.

### Method

#### Participants

Students (201 in total, 137 females), mean age = 24.3 years (*SD* = 4.29) participated. All were recruited by mail and social media invitations among students at a Norwegian University.

#### Material

##### Irrational Procrastination Scale (IPS)

This IPS (Steel, 2010) is a nine-item scale focusing on implemental delay (e.g., Item 7 “I delay tasks beyond what is reasonable”). It conforms to a unidimensional construct and demonstrates high internal consistency. In the present study, a translated and reduced six-item scale was used (Svartdal, 2017; Svartdal and Steel, 2017). Higher scores indicate increased procrastination. Internal reliability in the present sample was excellent, α = 0.93.

##### Negative Consequences of Procrastination (Custom)

As discussed, this scale aims to identify past NCP over different domains/situations. The scale should be administered immediately after a standard scale measuring procrastination to ensure that “procrastination” is understood in the same way across respondents. The format is open, with negative consequences indicated as examples. In the selection of examples, we explored potential examples from published literature, existing scale items, and face-valid examples. The actual examples selected were deemed to reflect possible NCP as perceived by students. Thus, this scale asks respondents to think back on situations in which planned and/or important tasks were delayed unnecessarily – “you procrastinated.” Then they were asked, with such situations in mind, to indicate (1–5, 1 = “does not fit at all” and 5 = “fits very well”) the appropriateness of eight assertions, with examples mentioned in parentheses. For example, item 1 addressed the social domain (negative reactions from others, e.g., that friends or acquaintances comment that I delay things unnecessarily), and item 3 addressed missed opportunities (e.g., that I did not respect an important deadline). For some domains/situations, two items were formulated. The full list of items and examples are listed in Appendix. We also included two additional items, one addressing stress (“The fact that I procrastinate gives me more stress”) and one addressing financial loss (“Putting things off till the last minute has cost me money in the past” (AIP item 15; McCown et al., 1989). These items were expected to overlap closely with NCP items 4 and 5.

##### Satisfaction With Life Scale (SWLS)

The five-item SWLS (Diener et al., 1985) measured life satisfaction. This scale aims to capture subjective global life satisfaction (e.g., item 3, “I am satisfied with my life”). Internal consistency in the present sample was α = 0.87.

##### Lack of Energy

Overall LoE was measured by a shortened version of the Chalder et al. (1993) LoE scale. This reduced version confirms to a single factor with good psychometric properties (DeArmond et al., 2014). Whereas the original scale asks respondents to rate LoE during the last 7 days, we reformulated the time span to “the last weeks.” An example item is “…how frequently have you felt physically exhausted.” Prior research in our group (unpublished) indicated that item 5 (“… had little or no desire to do anything”) produced a lower factor loading. This item was therefore not included in the present study. Internal consistency in the present sample was α = 0.90.

##### Social Loafing Tendencies

Self-reported social loafing was measured by five items from the Social Loafing Tendencies scale (Schippers, 2014). Sample items include “I prefer to let the other team members to do the work if possible” and “I contribute less than I should.” Internal consistency was good, α = 0.83.

#### Procedure and Ethics

All items were answered in a web-based survey (Qualtrics.com). Before answering, participants were briefly informed about the purpose of the study and actively consented to participate by pressing a button on the screen. The current study is part of a larger project that received ethical approval from the Regional Ethical Board in Tromsø, Norway (REK Nord 2014/2313) and the Ethical board of our university (December 2020). Participants were informed that they could participate in a lottery for a gift card by providing their phone number. This information was deleted prior to analysis.

#### Statistical Analysis

Exploratory factor analysis of the NCP scale was performed using the principal axis method. Prior to analysis, assumptions were tested by Bartlett’s test for sphericity and the Kaiser–Meyer–Olkin (KMO) measure of sampling adequacy. For these tests, as well as for computation of internal reliability in the scales used, we used Statistica 14.0.

Ordinary least squares (OLS) regression (robust standard errors) was used to assess the IPS and NCP in predicting the outcome variables. In these analyses, the IPS and NCP were first assessed separately for each outcome variable. In the second step, we examined the unique contribution of NCP in the explanation of dependent variables. However, as the NCP does not contain information on the level of procrastination, in the final step, we tested an adjusted NCP (i.e., NCP corrected for the individual’s level of procrastination). These analyses were performed in Stata 17.0.

### Results

Initial analyses indicated that age was not involved in any main or interactive effects. As for gender, men demonstrated significantly higher scores in the SWLS scores (*M* = 3.55 vs. 3.27, *p* = 0.02) and in the Social Loafing scores (*M* = 1.79 vs. 1.61, *p* = 0.04), whereas the LoE measure was significantly higher in women (*M* = 3.17 vs. 2.62, *p* < 0.001). However, no significant interaction effects were observed. Hence, the age and sex factors were not included in the analyses reported here.

#### Negative Consequences of Procrastination Scale

##### Basic Properties

Internal reliability (eight items) was good, α = 0.86. As this scale addresses negative consequences following procrastination, it should correlate moderately to highly with the IPS, and it did, *r* = 0.44 (see Table 2). As expected, the mean score of the NCP was lower compared to the IPS mean score, IPS mean = 2.99 vs. NCP mean 2.57 (Table 2).

**TABLE 2:** Descriptive statistics and Pearson’s correlations for study variables (*N* = 200).

Variables	Mean (*SD*)	(1)	(2)	(3)	(4)	(5)	(6)
(1) IPS	2.99(0.95)	1.000					
(2) NCP_all_	2.57(0.76)	0.443*	1.000				
(3) NCP_red_	2.67(0.84)	0.337*	0.957*	1.000			
(4) NCP_adj_	2.78(0.74)	0.830*	0.832*	0.795*	1.000		
(5) SWLS	3.34(0.76)	–0.167	−0.328*	−0.313*	−0.299*	1.000	
(6) LoE	3.03(0.93)	0.264*	0.389*	0.383*	0.398*	−0.532*	1.000
(7) S Loaf	1.68(0.64)	0.304*	0.408*	0.337*	0.396*	–0.200	0.029

NCP_*all*_, all NCP items included; NCP_*red*_, NCP with items 2, 3, 4, 6, 7; NCP_*adj*_, NCP adjusted by IPS; Correlations with *p < 0.005.

**FIGURE 1:**
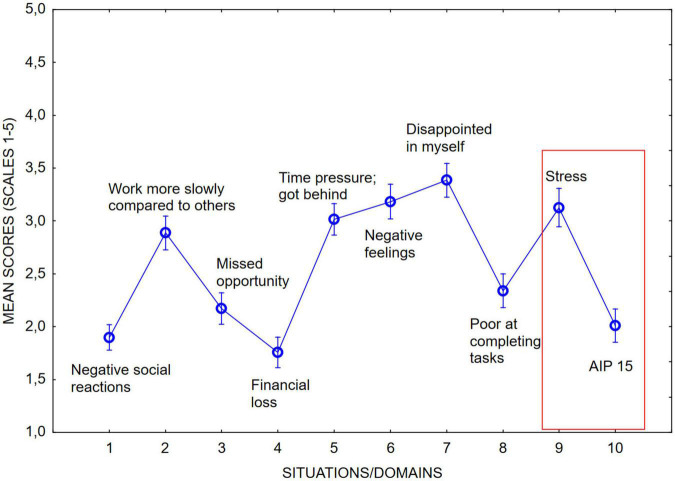
Mean scores of the NCP over different domains.

In the next step, we examined the occurrence of procrastination-related negative consequences over the different situations/domains probed. As is indicated in Figure 1, four indicators demonstrated higher scores compared to the others: Slow working pace compared to others, time pressure/getting behind, negative feelings, and disappointment of self. Further, as is apparent from the figure, the AIP 15 item corresponded well to the NCP item “Financial loss,” and the NCP item “Time pressure; got behind” corresponded well to the stress item. The correlation between IPS and the negative feelings item in NCP was *r* = 0.37, closely resembling the corresponding correlation reported by Lee and Hall (2020).

##### Dimensionality

An exploratory factor analysis (EFA) was conducted on the 8 NCP items with principal axis factoring extraction. The KMO measure of sampling adequacy was 0.84, well above the recommended minimum value of 0.5, and the Bartlett test of sphericity was significant [χ2 (28) = 732.81, *p* < 0.01] indicating suitability of the sample for factor analysis. The results indicated a unidimensional structure based on Scree plot test and examination of eigenvalues (greater than 1). The factor had eigenvalue of 3.58 and explained 44.7% of the variance. Factor loadings ranged from 0.48 (item 1) to 0.81 (items 6 and 7).

As the NCP was used in comparative analyses with the IPS (see next section), we also performed an EFA on the 8 NCP and 6 IPS items combined. The KMO measure of sampling adequacy was 0.90, and the Bartlett test of sphericity was significant [χ2(92) = 1736.12, *p* < 0.01], both indicating suitability of the sample for factor analysis. Results from the initial factor analysis produced two factors based on the Scree plot and eigenvalues greater than 1 (eigenvalues 4.60 and 3.37). However, items 1, 5, and 8 of the NCP demonstrated cross-loadings with the IPS factor and were deleted. A new iteration of EFA without NCP items 1, 5, 8 demonstrated two distinct constructs with no cross-loadings, eigenvalues 4.95 and 1.87. Factor loadings of the reduced NCP ranged from 0.40 (item 4, Financial loss) to 0.85 (item 7, Disappointment). The final structure model accounted for 61.96% of the variance. Internal reliability of this reduced NCP scale was good, α = 0.82.

#### Descriptive Results

Descriptive results are presented in Table 2. Of particular interest here is the lower mean score of the NCP (all items) compared to IPS, 2.57 vs. 2.99. This indicates that the overall NCP renders a more conservative estimate of procrastination problems compared to the IPS procrastination score, as expected. Also, note the moderate correlation between IPS and NCP, *r* = 0.44, indicating that these measures address similar but not identical constructs. Finally, note that the correlations between NCP and the scales measuring well-being, LoE, and social loafing were higher compared to the corresponding correlation to the IPS. As discussed, this probably reflects the fact that NCP addresses the maladaptive sides of procrastinatory episodes explicitly. These correlations also demonstrate predictable convergent as well as divergent validity for the NCP to established measures.

Two other versions of the NCP were computed. First, based on EFA results, we created a reduced NCP, the NCP_red_ (Items 1, 5, and 8 omitted). Note in Table 2 that the NCP_red_ correlates even lower to the IPS, *r* = 0.34. Second, because NCP does not contain information on procrastination level, a better measure might be suggested. Thus, we constructed an “adjusted” NCP, i.e., the NCP corrected for the individual’s level of procrastination. Different solutions were explored. The one reported here was calculated as the square root of the NCP score ^*^ IPS score. Hence, this adjusted index gives the same overall weight to the two scales. The resulting index, NCP_adj_, correlated highly with the IPS, *r* = 0.83, and 0.83 with the overall NCP score.

#### Regression Analyses

We performed separate regression analyses with social loafing, well-being, and LoE as dependent variables and IPS and NCP_red_ as predictors. The reduced version of NCP (five items, items 1, 5, and 8 excluded) was used. Here we expected that both predictors would explain the dependent variables. However, because the NCP addresses the problematic and maladaptive aspects of procrastination directly, it was expected to explain dependent measures better compared to IPS. The results confirmed these expectations. As is seen in Table 3, the NCP_red_ turned out to be a better predictor of all three dependent measures.

**TABLE 3:** Regression analysis (Beta/*R*^2^/robust SE) for IPS and NCP_*red*_ in predicting SWLS, LoE, and social loafing.

	IPS	NCP_red_
SWLS	−0.135/0.029/0.062*	−0.282/0.100/0.068
LoE	0.267/0.074/0.066	0.436/0.157/0.074
S Loafing	0.204/0.092/0.046	−0.257/0.114/0.060

NCP_red_ = items 1, 5, and 8 deleted. All effects = p < 0.001 except * < 0.05.

Second, we conducted separate hierarchical linear regression analyses to examine the unique contribution of NCP_red_ in the explanation of social loafing, well-being (SWLS), and LoE. Here, it was expected that NCP_red_ would significantly contribute to the explanation of the dependent variables. The results confirmed this expectation (see Table 4). IPS alone significantly contributed to the three regression models (i.e., Step 1) and accounted for 9.2, 2.9, and 7.4% of the variation in social loafing, SWLS, and LoE, respectively. Adding NCP_*red*_ into the model explained an additional 6.2, 7.5, and 10.3% of the variation in social loafing, SWLS, and LoE. This change (and the models with NCP_red_ term) was significant for all three variables. Further, as seen in Table 4, NCP_red_ was the most important predictor. In sum, this means that a measure of past NCP, the NCP_*red*_, contributes to explaining the dependent variables above the traditional IPS measure.

**TABLE 4:** Hierarchical regression analysis for SWLS, LoE, and social loafing.

Independent variables	SWLS		LoE		Social Loafing	
	β	Δ*R*^2^	β	Δ*R*^2^	β	Δ*R*^2^
**Step 1**						
IPS	-0.135*	0.029*	267**	0.074**	0.204**	0.092**
Adjusted *R*^2^	0.024		0.070		0.088	
**Step 2**						
IPS	–0.055		0.151*		0.144*	
NCP_*red*_	-0.260**	0.075**	0.377**	0.103**	0.201**	0.062**
*R* ^2^	0.104		0.178		0.154	
Adjusted *R*^2^	0.095		0.169		0.146	

*SWLS, subjective well-being; LoE, lack of energy; NCP_red_, reduced version of the NCP.*

*ΔR^2^ = R^2^ change. Unstandardized regression coefficients are reported. n = 197.*

*p < 0.05; **p < 0.001.

In the final step, we compared the IPS and NCP_adj_ by conducting linear regression analysis (performed separately for IPS and NCP_adj_). Since NCP_red_ does not contain information on procrastination level, IPS was compared with an adjusted version of NCP. Table 5 summarizes the results and shows that NCP_adj_, compared to IPS, was a better predictor and explained a larger proportion of variation in all dependent measures. These results indicate that the adjusted version was superior to the IPS, which supports our assumption about the importance of the NCP.

**TABLE 5:** Regression analysis (Beta/robust SE/*R*^2^) for IPS and IPS_*Adj*_ in predicting SWLS, LoE, and social loafing.

	IPS	NCP_adj_
SWLS	−0.135/0.062/0.029*	−0.306/0.083/0.092
LoE	0.267/0.066/0.074	0.515/0.081/0.169
S Loafing	0.204/0.046/0.092	0.343/0.064/0.157

NCP_adj_ = square root of NCP_red_ * IPS. All effects = p < 0.001 except *< 0.05.

In summary, the results supported our hypothesis that negative consequences of procrastination are important in making this form of delay detrimental. As discussed, not every type of delay is necessarily detrimental or procrastination (e.g., Klingsieck, 2013). Since the IPS scale is seemingly addressing both general delay and procrastination, it showed weaker relationships with subjective well-being, LoE, and social loading compared to the NCP_red_ and NCP_adj._

## Study 2

Study 2 was designed as a replication and extension of Study 1 with additional scales. First, we added a second procrastination scale, the implemental part of the Pure Procrastination Scale (PPS; Steel, 2010), as a supplement to the IPS. Second, three other scales supplemented those used in Study 1, the General Self-Efficacy Scale (GSE; Schwarzer and Jerusalem, 1995), a scale measuring negative thoughts and emotions in performance situations, the Achievement Motives Scale (AMS-R, five items covering negative emotions; Lang and Fries, 2006), and a scale assessing depression-like feelings and thoughts, the Behavioral Activation for Depression Scale (BADS-SF, short version; Kanter et al., 2007). Published research indicates that all three scales demonstrate predictable relationships with procrastination. Thus, self-efficacy is negatively related to procrastination, *r* = –0.44 (van Eerde, 2003), whereas procrastination correlates positively to a mastery-avoidance goal orientation (e.g., Howell and Watson, 2007) and to depression (van Eerde, 2003). The SWLS and LoE scales used in Study 1 were retained. Overall, we expected that the established procrastination scales, the IPS and PPS, would demonstrate predictable relationships with the dependent variables but that the NCP, especially the adjusted NCP, would perform better.

### Method

#### Participants

Students (223 in total, 180 females), mean age = 25.55 years (*SD* = 7.96) participated in the study. All were recruited by mail, social media, and lecture invitations among students at Norwegian universities. The relatively high proportion of females (ca. 80%) is somewhat higher compared to the overall proportion of females in the Norwegian student population (ca. 60%; Statistics Norway, 2021) but still typical of many study topics (e.g., psychology).

#### Material

Procrastination was measured by the IPS, as in Study 1. Also, the NCP (custom) scale, the SWLS (Diener et al., 1985), and the LoE (Chalder et al., 1993; DeArmond et al., 2014) scales were included, unchanged from Study 1. Detailed description of the scales is provided in Study 1, Methods section.

**Pure Procrastination Scale (Steel, 2010),** five middle items, were included. These items are all from the GPS (Lay, 1986) and are assumed to address implemental delay, as does the IPS (Svartdal and Steel, 2017). Example items are “In preparation for some deadlines, I often waste time by doing other things (PPS item 4; original GPS item 12) and “I generally delay before starting on work I have to do” (PPS item 8, original GPS 9). Higher PPS scores indicate increased procrastination. Internal reliability in the present sample was excellent, α = 0.94.

**Achievement Motives Scale (Lang and Fries, 2006**) has two subscales, one addressing approach motivation in achievement settings (e.g., “I like situations, in which I can find out how capable I am”), and one addressing avoidance motivation (called “fear of failure,” e.g., “If I do not understand a problem immediately I start feeling anxious”). In the present study, we included the five items addressing negative motivation. Lang and Fries (2006) reported good psychometric properties for the AMS-R, with CFA results supporting a two-factor structure in the general population (*N* = 3523) as well as in smaller student samples. In the present study, internal reliability for the 5-item subscale was good, α = 0.87.

**Behavioral Activation for Depression Scale (Short Form) (BADS-SF)**. The BADS-SF is a nine-item questionnaire designed to measure activation and avoidance tendencies associated with depression. It is based on a larger scale (Kanter et al., 2007). In the present study, we used eight items that load on the activation and avoidance subscales of the complete BADS (Kanter et al., 2007), e.g., “I engaged in many different activities” and “Most of what I did was to escape from or avoid something unpleasant.” The activation items were reversed so that the BADS-SF score reflected avoidance and less activation. Internal reliability in the present sample was acceptable, α = 0.73.

**General Self-efficacy Scale (Schwarzer and Jerusalem, 1995)** measured general self-efficacy beliefs (e.g., item 1, “I can always manage to solve difficult problems if I try hard enough”). Internal reliability for the GSE (10 items) was good in the present data set, α = 0.87.

#### Statistical Analysis

The statistical approach was identical to that used in Study 1.

### Results

Initial analyses indicated that age was not involved in any main or interactive effects. No sex difference was observed in the SWLS scores. Men demonstrated higher GSE scores compared to women (*M* = 3.54 vs. 3.88, *p* < 0.01), whereas women demonstrated higher scores on the BADS-SF subscale (*M* = 2.79 vs. 2.46, *p* < 0.01) and on the AMS-R subscale (*M* = 3.70 vs. 3.05, *p* < 0.01). As in Study 1, the LoE measure was significantly higher in women (*M* = 3.26 vs. 2.69, *p* < 0.01). However, no significant interaction effects were observed, and the variables (age, sex) were not included in subsequent analyses.

#### Negative Consequences of Procrastination Scale

Internal reliability in the NCP scale was good, α = 0.88. We conducted EFA (principal axis factoring, pomax rotation) with the same factor selection criteria as in Study 1. The results confirmed the results from Study 1, indicating the overall scale to confirm to a unidimensional construct, eigenvalue 3.80, 47% of the total variance accounted for. Adding the IPS and PPS into the exploratory factor analysis, the NCP items 1, 5, and 8 demonstrated cross loadings and were deleted. The remaining indicators conformed to two factors, eigenvalues 8.42 and 1.78, 63.78% of the total variance accounted for. These results repeated the outcomes from Study 1.

**FIGURE 2:**
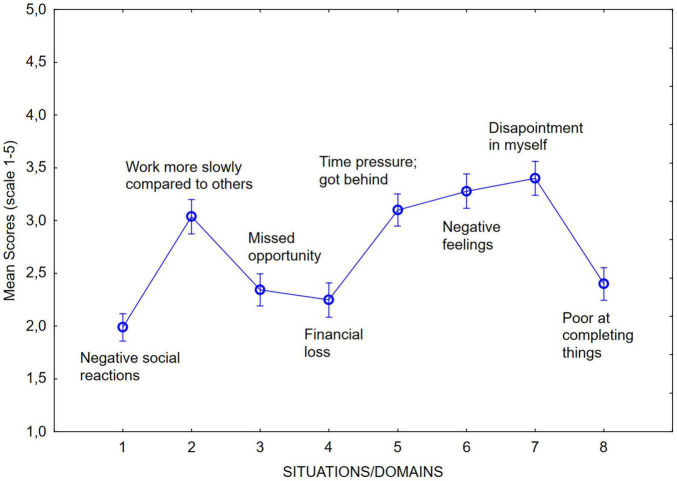
Mean scores of the NCP over different domains/situations.

Figure 2 displays the mean scores of negative consequences associated over the different situations/domains covered in the NCP. As in Study 1, four indicators demonstrated higher scores compared to the others: Slow working pace compared to others, time pressure/getting behind, negative feelings, and disappointment of self.

#### Descriptive Results

Means and correlations are shown in Table 6. Note again that NCP_*all*_ demonstrated a lower mean compared to the IPS and PPS, as was expected.

**TABLE 6:** Descriptive statistics and Pearson’s correlations for study variables (*N* = 222).

	*M* (*SD*)	(1)	(2)	(3)	(4)	(5)	(6)	(7)	(8)	(9)	(10)
(1) IPS	3.07 (0.93)	1.000									
(2) PPS	3.03 (1.13)	0.845	1.000								
(3) NCP_all_	2.72 (0.86)	0.517	0.521	1.000							
(4) NCP_red_	2.86 (0.96)	0.449	0.456	0.970	1.000						
(5) NCP_adj_IPS	2.92 (0.81)	0.839	0.760	0.875	0.856	1.000					
(6) NCP_adj_PPS	2.89 (0.90)	0.781	0.876	0.845	0.819	0.947	1.000				
(7) SWLS	3.44 (0.72)	–0.329	–0.325	–0.372	–0.373	–0.413	–0.411	1.000			
(8) LoE	3.14 (0.94)	0.316	0.309	0.293	0.324	0.374	0.371	–0.457	1.000		
(9) AMS-R	3.55 (0.97)	0.218	0.272	0.441	0.464	0.403	0.413	–0.392	0.486	1.000	
(10) GSE	3.61 (0.58)	–0.220	–0.246	–0.420	–0.393	–0.372	–0.375	0.439	–0.280	–0.503	1.000
(11) BADS	2.72 (0.67)	0.508	0.492	0.504	0.476	0.585	0.582	–0.504	0.571	0.504	–0.503

*NCP_all_, all NCP items included; NCP_red_, NCP with items 2, 3, 4, 6, 7; NCP_adj_IPS, NCP adjusted by IPS; NCP_adj_PPS, NCP adjusted by PPS.*

*All correlations p < 0.005.*

#### Regression Analyses

Table 7 displays the beta values as well as R^2^ in predictions of the dependent measures of Study 2. In these analyses, the IPS, PPS, and NCP were entered in separate regression analyses. Note that the NCP_*red*_ demonstrated similar predictive abilities as the procrastination scales for SWLS, LoE, and BADS-SF, and better for AMS-FF and GSE.

**TABLE 7:** Regression analysis (Beta/*R*^2^) for IPS and PPS in predicting SWLS, LoE, AMS-FF, GSE, and BADS-SF.

	IPS	PPS	NCP_red_
SWLS	–0.255/0.108	–0.208/0.106	–0.282/0.139
LoE	0.317/0.097	0.245/0.087	0.315/0.101
AMS-FF	0.225/0.046	0.224/0.068	0.468/0.210
GSE	–0.137/0.048	–0.125/0.060	–0.238/0.154
BADS-SF	0.365/0.256	0.287/0.234	0.333/0.224

*NCP_red_ = NCP with items 2, 3, 4, 6, 7.*

Second, we conducted separate hierarchical linear regression analyses to examine the unique contribution of NCP_*red*_ in the explanation of well-being (SWLS), LoE, fear of failure (AMS-FF), general self-efficacy (GSE), and depression-related behavioral activation (BADS-FF). Here it was expected that NCP_red_ would significantly contribute to the explanation of the dependent variables. The results confirmed this expectation. The results for IPS are displayed in Table 8. IPS alone significantly contributed to the three regression models (i.e., Step 1) and accounted for 10.8, 9.7, 4.6, 4.8, and 6.9% of the variation in SWLS, LoE, AMS-FF, GSE, and BADS-SF, respectively. Adding NCP_red_, the model explained an additional 6.4, 3.9, 16.3, 10.8, and 4.7% of the variation in SWLS, LoE, AMS-FF, GSE, and BADS-FF. This change (and the models with NCP_red_ term) was significant for all variables. Further, as seen in Table 8, NCP_red_ was the most important predictor (except LoE where IPS and NCP_red_ were equal). Similar results were observed for the second procrastination scale, PPS (see Table 9). In sum, these results indicate that the past negative consequences of procrastination measure (NCP) contributed to explaining the dependent variables above traditional procrastination measures, the IPS and PPS.

**TABLE 8:** Hierarchical regression analysis for SWLS, LoE, AMS-FF, GSE, and BADS-SF.

Independent variables	SWLS	LoE	AMS-FF	GSE	BADS-SF
	**β**	**β**	**β**	**β**	**β**

**Step 1**					
IPS	-0.255**	0.317**	0.225*	-0.137*	0.365**
Adjusted *R*^2^	0.104	0.093	0.042	0.044	0.253
Δ*R*^2^	0.108**	0.097**	0.046*	0.048*	0.256**
**Step 2**					
IPS	-0.157*	0.215*	0.013	–0.034	0.266**
NCP_*red*_	-0.213**	0.220*	0.462**	-0.223**	0.217**
*R* ^2^	0.172	0.137	0.210	0.156	0.332
Adjusted *R*^2^	0.164	0.129	0.202	0.149	0.326
Δ*R*^2^	0.064**	0.039**	0.163**	0.108**	0.078**

*SWLS, subjective well-being; LoE, lack of energy; AMS-FF, achievement motivation scale – fear of failure; GSE, general self-efficacy; BADS-SF, behavioral activation for depression scale – short form; NCP_red_, reduced version of NCP.*

*ΔR^2^ = R^2^ change. Unstandardized regression coefficients are reported. n = 223.*

*p < 0.05; **p < 0.001.

**TABLE 9:** Hierarchical regression analysis for SWLS, LoE, AMS-FF, GSE, and BADS-SF.

Independent variables	SWLS	LoE	AMS-FF	GSE	BADS-SF
	**β**	**β**	**β**	**β**	**β**

**Step 1**					
PPS	-0.208**	0.245**	0.224**	-0.125*	0.287**
Adjusted *R*^2^	0.102	0.083	0.063	0.055	0.231
Δ*R*^2^	0.106**	0.087**	0.068**	0.060**	0.234**
**Step 2**					
PPS	-0.125*	0.157*	0.055	–0.042	0.201**
NCP_red_	-0.215**	0.229*	0.438**	-0.215**	0.224**
*R* ^2^	0.169	0.129	0.213	0.159	0.315
Adjusted *R*^2^	0.162	0.121	0.201	0.151	0.308
Δ*R*^2^	0.064**	0.042*	0.145**	0.100**	0.080**

*SWLS, subjective well-being; LoE, lack of energy; AMS-FF, achievement motivation scale – fear of failure; GSE, general self-efficacy; BADS-SF, behavioral activation for depression scale – short form; NCP_red_, reduced version of NCP.*

*ΔR^2^ = R^2^ change. Unstandardized regression coefficients are reported. n = 223.*

*p < 0.05; **p < 0.001.

Finally, we compared the IPS, PPS, and NCP_adj_ by conducting linear regression analysis (performed separately for IPS, PPS, and NCP_adj_). Since NCP_red_ does not contain information on the level of procrastination, IPS and PPS were compared with adjusted versions of NCP. Here, two adjusted versions were created, one using the IPS and one using the PPS. Table 10 summarizes the results and demonstrates that the NCP_adj_, in comparison to both the IPS and PPS, was a better predictor and explained a larger proportion of variation in all dependent measures. Both adjusted versions of NCP performed better than their original counterparts. These results indicate that adjusted versions are superior to IPS and PPS, supporting the results of Study 1 and also our assumptions about limited importance of NCP.

**TABLE 10:** Regression analysis (Beta/robust SE/*R*^2^) for IPS and IPS_Adj_ in predicting SWLS, LoE, GSE, and BADS-SF.

	IPS	NCP_adjIPS_	PPS	NCP_adjPPS_
**SWLS**	−0.255/0.052/0.108	−0.366/0.053/0.171	−0.208/0.042/0.106	−0.329/0.049/0.169
**LoE**	0.317/0.068/0.097	0.428/0.072/0.136	0.245/0.062/0.087	0.375/0.071/0.129
**AMS-FF**	0.225/0.074/0.046*	0.477/0.072/0.158	0.224/0.060/0.68	0.434/0.067/0.162
**GSE**	−0.137/0.041/0.048	−0.265/0.041/0.138	−0.125/0.032/0.060	−0.239/0.038/0.139
**BADS-SF**	0.365/0.039/0.256	0.482/0.038/0.340	0.287/0.036/0.234	0.428/0.036/0.331

NCP_*adj*_, square root of NCP_*red*_ * IPS/PPS. All effects = *p* < 0.001 except * ≤ 0.05.

## General Discussion

Delays may be rational and functional, but sometimes dysfunctional and irrational. The delays seen in procrastination are, by definition, irrational. A common definitional criterion is that procrastination is characterized by delaying “despite expecting to be worse off for the delay” and that the procrastinating individual “acts against better judgment.” Examination of common procrastination scales demonstrates, however, that scales do not address these core characteristic of procrastination. In effect, there is a gap between the definition of procrastination and how it is measured in common scales.

Accordingly, the present paper explored the utility of a brief scale, the NCP, to supplement existing scales. Assuming that subjective past negative consequences of procrastination reflect the maladaptive and irrational aspects of this habit,^4^ this scale should capture these aspects better than traditional scales. The NCP probes negative consequences of procrastinatory episodes over different domains and situations, as well as negative emotions and cognitions associated with such episodes, thus capturing a broad spectrum of troublesome sides associated with procrastination. In two studies, we demonstrated that this scale seems to tap the maladaptive aspects of unnecessary delay better than standard procrastination scales, here the IPS and PPS. Specifically, common procrastination scales demonstrate predictable negative relationships to scales measuring positive states (e.g., well-being, self-efficacy) and reliable positive relationships to scales measuring negative states (e.g., LoE). Comparing the NCP to established procrastination scales in the prediction of well-being, social loafing, and LoE (Study 1) and well-being, general self-efficacy, LoE, negative motivation, and mild depression tendencies (Study 2), the NCP seemed to be superior in predictions. Importantly, as the NCP identifies problems associated with past procrastination but does not itself contain information on procrastination levels, an improved NCP score is achieved by adjusting it for individual procrastination levels. We explored different ways of performing this adjustment. A simple approach is to adjust by using an established procrastination scale, here IPS or PPS. This alternative was systematically explored, and for every comparison, the adjusted NCP, the NCP_*adj*_, outperformed the IPS and PPS in predictive ability.

An important implication of the present studies is that the NCP seems to capture a critical feature of procrastination, breakdown in self-regulation (e.g., Steel, 2007), better than traditional scales. As failure to self-regulate is associated with negative emotions (Tice and Bratslavsky, 2000; Tice et al., 2001), discomfort and negative cognitions/motions associated with procrastinatory episodes may be important criteria for maladaptive and irrational delays. This also applies to situations where the individual may achieve temporary emotional benefits from avoiding or escaping aversive work, as in short-term mood repair. Short-time mood repair is itself a sign of a breakdown in self-regulation, and negative emotions return (e.g., Sirois and Pychyl, 2013). Given that negative consequences – discomfort and negative cognitions/emotions – are connected intimately to a core problem in procrastination, failure to self-regulate, the NCP provides a simple means of capturing that core. Of note, whereas prior research has linked procrastination to negative affect in a general way (e.g., Krause and Freund, 2014), the present studies obtained a measure of discomfort and negative affect/cognition to procrastinatory episodes specifically.

By focusing on past NCP rather than forward-looking expectations, it may appear that we underestimate the ability of procrastinating individuals to assess their own procrastinatory behavior. We do not. First, research has amply documented that procrastinators are aware of their procrastination, both as a general dysfunctional habit (e.g., Steel, 2010) and in dealing with specific tasks (e.g., Tuckman, 1991). Second, we agree with the general definition of procrastination as a maladaptive delay in planned behavior, given the individual’s own standard. Both criteria indicate that the procrastinating individual cognitively is capable of making plans as well as evaluating the factual progression in (not) realizing them. In fact, a core problem of procrastination is that those insights do not propel the individual to get things done. This problem, often named the intention-action gap (Steel et al., 2001; Steel, 2010), addresses implemental delays. The procrastination scales used in the present studies, the IPS and the PPS (Steel, 2010), focus on implemental delay. However, even these turned out to be rather indiscriminate in measuring procrastination, probably because they address delays in different forms, including those intention-action gaps that are trivial and inconsequential. The retrospective measure of the maladaptive consequences of procrastination explored in the present paper seems to demonstrate better construct validity. Overall, procrastination seems to account for only a limited amount of variance in the scales explored in the present studies. Still, that proportion seems to be measured more appropriately by the NCP, and especially the adjusted NCP, as this scale accounted for a larger proportion of the explained variance when compared to standard procrastination scales.

The NCP scale may be helpful in research as well as applied purposes. Self-report scales are often criticized, with behavioral procrastination measures (Miyake and Kane, 2021) or momentary assessment of procrastination in experience sampling (Wieland et al., 2018) suggested as better alternatives. However, the present results indicate an important advantage of self-report measures of procrastination over behavioral measures in that they, in a unique way, address the subjective criteria for problematic and irrational delays as distinct from delays that are unproblematic (e.g., Krause and Freund, 2014). Here, the NCP may help differentiate those forms of procrastination that reflect a maladaptive style of life from delays that are unnecessary but still inconsequential. As Díaz-Morales and Ferrari (2015) put it: “*Everyone procrastinates, but not everyone is a procrastinator”* (p. 308, italics in original). Hence, an implication of the current studies is that unnecessary delay *per se* may not be as troublesome as having a history with procrastination-related negative consequences. The NCP seems to be capable of identifying (at least indirectly) the maladaptive and irrational sides of the procrastination habit.

The NCP may be a helpful tool in preventive as well as in clinical/applied settings. As for prevention, this scale may provide information on when and where procrastination has become problematic, thus helping educators and counsellors identify contexts that are especially appropriate for preventive measures. For example, if procrastination is especially problematic in the social domain, preventive measures may focus on social factors in the study environment (e.g., Codina et al., 2020). In clinical applications, the NCP scale may be a useful tool also. As discussed, not all delay is problematic. For example, Rozental et al. (2015) identified five main groups or clusters of procrastinators, with only 33% of participants representing severe instances of procrastination potentially requiring tailored treatment interventions. As procrastination-related negative feelings and cognitions are markers of problematic delay, the NCP may represent a good utility in clinical contexts where more precise information on problematic procrastination is needed. Also, the scale might be used to screen for participants’ levels of problematic procrastination before entering a clinical trial or in creating create groups when conducting analyses to study the efficacy of interventions to reduce procrastination. In such cases, there is a need to distinguish general procrastination (as measured by standard scales) from maladaptive forms (as measured by the NCP). Also, as the NCP is capable of connecting maladaptive delays to specific domains/situations, interventions may be adapted to individual procrastination profiles in ways not possible when using standard scales. Here, the results from a study on academic procrastination by Steel and Klingsieck (2016) are particularly relevant. These authors identified a common factor important for all procrastinators, conscientiousness (and its facets, e.g., self-discipline, impulsiveness). However, after controlling for conscientiousness, students appeared to procrastinate for different reasons. For example, some procrastinated for social reasons (those high in extraversion), whereas others put off because of anxiety (those high in neuroticism). The NCP presents itself as a simple tool to identify individual procrastination profiles, which in turn may be of great utility in creating tailor-made interventions and assessing their effects.

Another important distinction relates to forms of delay that are rational and necessary for optimal goal-striving (e.g., strategic delay; Klingsieck, 2013). As discussed in this paper, commonly used procrastination scales do not contain items that explicitly differentiate trivial forms of delay from problematic delays. In addition, existing scales also often fail in identifying rational forms of delay as distinct from procrastination. Sometimes delayed action may be rational and even necessary for optimal goal striving. For example, a student might feel ready to submit her thesis in good time before the deadline, but submitting too early (precrastination) might induce unexpected costs in terms of lower quality and errors. Hence, delaying planned submission may be the rational thing to do. In general, timely action is important, both for the individual, for people interacting with the individual, and for society in general. When timely action is delayed (or rushed), negative consequences are likely to appear. Accordingly, although there may be somewhat blurred boundaries between them, at least three forms of delay should be differentiated:

(1) **Strategic and rational delays**. Delays that are *rational and often necessary for optimal goal striving* (e.g., delay submitting your thesis because your supervisor asked you to rewrite the discussion part).
(2) **Inconsequential delay**. Delays that are *unnecessary but bear no negative consequences* (e.g., reading a chapter on Wednesday rather than on Tuesday as preparation for a lecture on Friday).
(3) **Irrational delay**. Delays that are *maladaptive, given an intended goal* (e.g., not reading a chapter before a lecture even though the teacher strongly recommended you to).

The scale presented in the present paper, the NCP, seems to be capable of separating the two latter. Thus, a simple measure focusing on past negative consequences of unnecessary delay may be useful in separating trivial forms of unnecessary delay (2 above) from more severe forms (3 above). For research purposes, the NCP should be adjusted by a validated procrastination scale.

### Limitations and Future Research

Several limitations of the present studies should be noted. First, as participants in the present studies were students only, using convenience samples, the next step would be to explore the NCP in a sample from the general population. Here, the item examples in the scale should be carefully examined, ensuring that they also reflect problematic consequences as perceived by non-students. Such a study should also apply more stringent methods to assess the scale, such as confirmatory factor analysis (CFA) and item response theory (IRT). Second, the sample sizes of the present studies may be considered as a limitation. Still, based on minimum sample size criteria for EFA (e.g., Kyriazos, 2018) and cross-validation of the factorial structure (Study 2), the NCP has acceptable empirical support. Nevertheless, future studies are advised to repeat the study with a larger sample. Third, the situations and domains probed in the present studies should be thoroughly examined. We selected a relatively broad spectrum of situations and domains to tap negative consequences of unnecessary delay, but other domains might be included. For example, we did not probe the health domain (e.g., Sirois and Pychyl, 2018), which is potentially important, especially as people get older. On the other hand, the present data indicate that even a reduced scale functioned very well, indicating that the scale might work well even if probes are taken from only a limited set of domains/situations. Fourth, given the broad classes of delay discussed in the previous section, a further step forward might be to develop a brief scale that probes the tendency to delay strategically and rationally. To the best of our knowledge, there is currently no scale that measures the first broad class mentioned, the tendency to delay things that are rational and often necessary for optimal goal striving. Such a scale could complement the NCP in helping to achieve an even more precise delimitation of the phenomena we call procrastination.

## Conclusion

The present paper explored the utility of a brief scale to measure past negative consequences associated with procrastinatory episodes. This scale seems to be helpful in separating trivial forms of unnecessary delay from maladaptive forms and helps identify the core problem in procrastination, maladaptive and irrational delay. As such, this scale represents a potentially valuable tool in research and clinical/applied efforts.

## Data Availability Statement

The raw data supporting the conclusions of this article will be made available by the authors, without undue reservation.

## Ethics Statement

The studies involving human participants were reviewed and approved by the Department of Psychology, Ethics Committee. The participants provided their written informed consent to participate in this study.

## Author Contributions

FS initiated the project, drafted the manuscript, collected the data, and conducted the statistical analyses. EN contributed to the statistical analyses and overall refinement of the manuscript. Both authors edited the complete draft.

## Conflict of Interest

The authors declare that the research was conducted in the absence of any commercial or financial relationships that could be construed as a potential conflict of interest.

## Publisher’s Note

All claims expressed in this article are solely those of the authors and do not necessarily represent those of their affiliated organizations, or those of the publisher, the editors and the reviewers. Any product that may be evaluated in this article, or claim that may be made by its manufacturer, is not guaranteed or endorsed by the publisher.

## Footnotes

1 “Discomfort” or “negative consequences” refer to subjective discomfort (e.g., Solomon and Rothblum, 1984; Ferrari, 1998) or other negative consequences (e.g., Simpson and Pychyl, 2009).

2 Note that future thought as used in the Stainton study were induced by researchers and were not independently generated by participants. Thus, these findings do not contradict our previous argument on subjective availability of future negative consequences.

3 In the present scale development, the authors and a student group discussed domains/situations and possible scale items.

4 See Grund and Fries (2018) for a recently proposed alternative explanation.

## Appendix

### The NCP Scale

Think of situations where you have put off planned and/or important tasks unnecessarily – you have “procrastinated.”

When I think back on such situations, I must say that my procrastination resulted in…

…negative reactions from others (for example, that others remarked that I put off things unnecessarily).

…I perceived myself as inferior to others (for example, that others worked much faster than I did and finished tasks long before I did).

…that I missed opportunities (for example that I missed an important deadline).

…that I have lost something by being late (for example, that I was late in paying a bill and got a big fine).

…that I experienced lack of time or got behind (for example, that I did not read a recommended chapter before a lecture so that I did not understand the lecture).

…that I experienced negative feelings (for example, shame, regret, guilt, or worry).

…that I was disappointed in myself (for example, that I had expected to accomplish what I had planned but failed).

…that my belief that I am not good at finishing things was confirmed.
